# Overexpression of the vitamin D receptor (VDR) induces skeletal muscle hypertrophy

**DOI:** 10.1016/j.molmet.2020.101059

**Published:** 2020-08-07

**Authors:** Joseph J. Bass, Asif Nakhuda, Colleen S. Deane, Matthew S. Brook, Daniel J. Wilkinson, Bethan E. Phillips, Andrew Philp, Janelle Tarum, Fawzi Kadi, Ditte Andersen, Amadeo Muñoz Garcia, Ken Smith, Iain J. Gallagher, Nathaniel J. Szewczyk, Mark E. Cleasby, Philip J. Atherton

**Affiliations:** 1 MRC/ARUK Centre for Musculoskeletal Ageing Research and National Institute for Health Research (NIHR), Nottingham Biomedical Research Centre (BRC), School of Medicine, University of Nottingham, DE22 3DT, UK; 2 Department of Sport and Health Sciences, University of Exeter, EX1 2LU, UK; 3 Mitochondrial Metabolism and Ageing Laboratory, Diabetes and Metabolism Division, Garvan Institute of Medical Research, NSW, 2010, Australia; 4 School of Sport, Exercise and Rehabilitation Sciences, University of Birmingham, B15 2TT, UK; 5 School of Health Sciences, Örebro University, 70182, Sweden; 6 Molecular Physiology of Diabetes Laboratory, Dept. of Comparative Biomedical Sciences, Royal Veterinary College, NW1 0TU, UK; 7 Institute of Metabolism and Systems Research, The University of Birmingham, Birmingham, UK; 8 Department of Bioinformatics – BiGCaT, NUTRIM School of Nutrition and Metabolism in Translational Research, Maastricht University, Maastricht, the Netherlands; 9 Physiology, Exercise and Nutrition Research Group, Faculty of Health Sciences and Sport, University of Stirling, FK9 4LA, UK

**Keywords:** Vitamin D, Skeletal muscle, Metabolism, Exercise

## Abstract

**Objective:**

The Vitamin D receptor (VDR) has been positively associated with skeletal muscle mass, function and regeneration. Mechanistic studies have focused on the loss of the receptor, with *in vivo* whole-body knockout models demonstrating reduced myofibre size and function and impaired muscle development. To understand the mechanistic role upregulation of the VDR elicits in muscle mass/health, we studied the impact of VDR over-expression (OE) *in vivo* before exploring the importance of VDR expression upon muscle hypertrophy in humans.

**Methods:**

Wistar rats underwent *in vivo* electrotransfer (IVE) to overexpress the VDR in the *Tibialis anterior* (TA) muscle for 10 days, before comprehensive physiological and metabolic profiling to characterise the influence of VDR-OE on muscle protein synthesis (MPS), anabolic signalling and satellite cell activity. Stable isotope tracer (D_2_O) techniques were used to assess sub-fraction protein synthesis, alongside RNA-Seq analysis. Finally, human participants underwent 20 wks of resistance exercise training, with body composition and transcriptomic analysis.

**Results:**

Muscle VDR-OE yielded total protein and RNA accretion, manifesting in increased myofibre area, i.e., hypertrophy. The observed increases in MPS were associated with enhanced anabolic signalling, reflecting translational efficiency (e.g., mammalian target of rapamycin (mTOR-signalling), with no effects upon protein breakdown markers being observed. Additionally, RNA-Seq illustrated marked extracellular matrix (ECM) remodelling, while satellite cell content, markers of proliferation and associated cell-cycled related gene-sets were upregulated. Finally, induction of VDR mRNA correlated with muscle hypertrophy in humans following long-term resistance exercise type training.

**Conclusion:**

VDR-OE stimulates muscle hypertrophy ostensibly via heightened protein synthesis, translational efficiency, ribosomal expansion and upregulation of ECM remodelling-related gene-sets. Furthermore, VDR expression is a robust marker of the hypertrophic response to resistance exercise in humans. The VDR is a viable target of muscle maintenance through testable Vitamin D molecules, as active molecules and analogues.

## Highlights

• Increased vitamin D receptor (VDR) expression stimulates skeletal muscle hypertrophy.
• Anabolic signalling, ribosomal biogenesis, and muscle protein synthesis are enhanced.
• Coordinated upregulation of extracellular remodelling related gene-sets.
• VDR expression correlates with resistance exercise induced hypertrophy in humans.

## 1 Introduction

The classical function of Vitamin D is to regulate calcium (Ca^2+^) and phosphate (P_i_) homeostasis to maintain bone health and prevent bone-related disorders [1,2]. However, Vitamin D influences tissues other than bone, acting through its ubiquitously expressed Vitamin D receptor (VDR) [3], with exogenous Vitamin D upregulating VDR mRNA and Vitamin D metabolism in skeletal muscle [4]. Studies have demonstrated Vitamin D deficiency results in a reduction in skeletal muscle mass and function [5]; conversely, vitamin D supplementation enhances muscle function and myofibre cross-sectional area (CSA) in the elderly [6,7] and athletes [8,9]. Despite previous controversy regarding the presence of the VDR in skeletal muscle, recent studies have confirmed expression of the VDR [10,11]. Furthermore, CYP27B1 (responsible for conversion of inactive 25-hydroxyvitamin D_3_ to active 1,25-dihydroxyvitamin D_3_) is present in fully differentiated skeletal muscle of both rodents and humans [12]. The VDR is a nuclear receptor, binding to promotors of specific DNA sequences at Vitamin D-responsive elements (VDREs) to regulate transcription of Vitamin D-responsive genes [13]. Furthermore, non-transcriptional VDR signalling has also been revealed [14] and may be functionally regulated through post-translational modifications [15].

Ostensibly autonomous roles of the VDR in skeletal muscle (i.e., independent from Vitamin D) have also been reported, with decreased VDR protein expression being linked to multiple disease states and ageing [16,17]. Notably, multiple studies have explored the implications of loss of the VDR, primarily in whole-body knockout animal models, with reductions in muscle fibre size and strength being observed [18,19]. Likewise, transient muscle-specific VDR knockdown impairs myogenic differentiation [20], and VDR expression is required for Vitamin D-induced anti-proliferative/pro-differentiation effects in myoblasts [21]. In clinical studies, VDR expression is elevated and linked to regeneration following muscle damage [12] and is acutely upregulated (1–3 h) following resistance exercise [22], indicating a link to muscle growth and maintenance. However, despite pre-clinical and clinical suggestions of a link between muscle VDR expression and muscle mass/function, no studies have investigated the mechanistic or functional role of increased VDR expression in skeletal muscle.

Therefore, the aim of this study was to explore the mechanistic role of the VDR in skeletal muscle. To achieve this, we locally overexpressed the VDR *in vivo* using electrotransfer techniques; in doing so, we observed myofibre hypertrophy, upregulated mTOR signalling, global muscle protein synthesis (MPS) and satellite cell recruitment. Finally, we validated the clinical relevance of this by showing that upregulation of VDR pathway expression is positively associated with muscle hypertrophy following resistance training in humans.

## 2 Materials and methods

### 2.1 Animal handling

All animal experimental procedures were undertaken and approved by the Royal Veterinary College's Ethics and Welfare committee and carried out under UK Home Office licence to comply with the Animals (Scientific Procedures) Act 1986. Eight-week-old male Wistar rats were housed at 22 ± 0.5 °C under a 12 h day/12 h night cycle and acclimatised to their new surroundings for one week. Animals were fed a normal maintenance chow diet *ad libitum* (Special Diet Services, LBS Biotechnology, Surry, UK) (with standard Vitamin D_3_ fortification at 621.7 IU/kg to prevent deficiency). Ten days after *in vivo* electrotransfer (IVE), all rats were fasted overnight and underwent euthanasia by injection of pentobarbitone. Animals were fasted to determine “basal” anabolic signalling responses separate from growth conditions (i.e., fed response). A sub-set of animals underwent an intraperitoneal glucose tolerance test (IPGTT) combined with administration of 2-[1,2–^3^H(*N*)]-deoxy-d-glucose tracer (Perkin–Elmer; Seer Green, Bucks, UK) to assess muscle glucose uptake and glycogen content as previously described [23]. Blood was collected by tail nick at 0, 15, 30, 60 and 90 min post glucose injection and measured immediately using an Accu-Check Advantage meter (Roche Diagnostics, Burgess Hill, West Sussex, UK). Muscles were rapidly dissected after euthanasia; transverse sections were mounted on cork tiles in optimum cutting temperature (OCT) medium and snap-frozen in liquid nitrogen-cooled isopentane. The remaining muscle was freeze-clamped and stored at −80^°^C. For glucose uptake into muscles, plasma at each time point was deproteinised and radioactivity determined by liquid scintillation counting in Ultima Gold XR (Perkin–Elmer) on an LS6500 Multipurpose scintillation counter (Beckman Coulter, High Wycombe, UK) before the area under the curve (AUC) was calculated. Frozen muscle was powdered and homogenised in water and phosphorylated deoxyglucose separated by an anion exchange resin Bio-Rad Laboratories, Hemel Hempstead, UK) before β-scintillation counting to calculate the clearance of deoxyglucose into each muscle [23].

### 2.2 *In vivo* electrotransfer (IVE)

IVE procedures were undertaken as previously described [24]. Briefly, animals were anaesthetised using isofluorane (2.5%) and their hind limbs shaved and prepared with ethanol. *Tibialis anterior* muscles (TAM) were injected intramuscularly with six spaced 50-μl aliquots of lenti-shRNA particles prepared in endotoxin-free sterile saline at 0.5 mg/ml. TAMs were chosen due to their relatively superficial nature and previously validated ability to overexpress specific proteins through IVE genetic manipulation [24]. Animals received pCMV6-mVDR (Origene, MR206711) into the right and empty pCMV6 controls into the left TAM. Immediately following this, one 900 V/cm, 100 μs pulse and four 90 V/cm, 100 ms pulses were administered across the distal limb via tweezer-electrodes attached to an ECM-830 electroporator (BTX, Holliston, MA). Animals subsequently received a subcutaneous injection of carprofen (50 mg/kg) before recovery from anaesthesia.

### 2.3 Measurement of muscle protein synthesis (MPS)

Seven days post-IVE, animals received a D_2_O bolus through oral gavage (7.2 ml/kg, 70 atom %) to measure cumulative MPS. To determine peak D_2_O body water enrichment, 2 animals were euthanised 2 h after oral gavage, and blood was collected in pre-chilled tubes containing lithium heparin. These were subsequently cold centrifuged at 1,750 g, with plasma fractions separated into aliquots and frozen at −80^°^C. Ten days after IVE, animals were overnight fasted and euthanised before blood and muscle was collected.

Isolation of myofibrillar, mitochondrial, cytoplasmic and collagen protein was undertaken as previously described [25]. Briefly, 50 mg of muscle was homogenised in ice cold homogenisation buffer, before continuous vortexing for 10 min and centrifugation at 13,000 g for 5 min at 4^°^C, and sarcoplasmic protein containing supernatant was collected and precipitated in 1 M of perchloric acid (PCA). The pellet was further homogenised using an ice cold dounce homogeniser in cold mitochondrial extraction buffer (MEB) before centrifugation at 1,000 g for 5 min at 4°C and supernatant collection. Pelleted myofibrillar proteins were solubilised in 0.3 M of NaOH at 37 °C for 30 min. Collagen was pelleted by centrifugation at 13,000 g for 10 min, and myofibrillar containing supernatant was removed and precipitated in 1 M of PCA. Pellets were washed in 70% ethanol, before being hydrolysed overnight at 110 °C in 1 ml of 0.1 M HCl and 1 ml of H^+^ Dowex resin.

Hydrolysed amino acids were eluted into 2 M of NH4OH then evaporated to dryness. Deuterium labelling of protein-bound alanine was determined though conversion to its tert-butyldimethysilyl derivative and assessed by single ion monitoring (SIM) of m/z 260 and 261 by gas chromatography-mass spectrometry. D_2_O enrichment of plasma was measured using a modified acetone exchange method [26]. Two microliters of 10N NaOH and 1 μl of acetone was added to 100 μl of plasma and vortex mixed for 15 s. Following 24 h of incubation at room temperature, to allow the exchange of hydrogen atoms for deuterium, acetone was extracted into n-heptane and injected into a gas chromatograph-mass spectrophotometer. D_2_O enrichment was measured via SIM of m/z 58 and 59 referenced to a standard curve of known D_2_O enrichments. The fractional synthetic rate (FSR) was calculated using the following equation:

FSR (%/h) = [(MPE_Ala_)]/[3.7× (MPE_MW_) x *t* ] x 100

MPE_Ala_ represents protein-bound alanine enrichment, MPE_MW_ represents plasma water enrichment (corrected for mean number of deuterium moieties incorporated per alanine, 3.7) and *t* signifies time in hours [27].

### 2.4 qRT-PCR

RNA from skeletal muscle was extracted into TRizol reagent (Invitrogen 15596026) and reverse transcribed using a High-Capacity cDNA Reverse Transcription kit (Applied Biosystems 4368814), both following the manufacturer's recommendations. qRT-PCR was performed using SYBR Select Master Mix (Applied Biosystems 4472908) with primers designed in-house using Primer Express® (Table S1) on a ViiA®7 Real-Time PCR system in triplicate (Life Technologies). Quantification was performed using the 2^−ΔCT^ method and normalised to glyceraldehyde 3-phosphate dehydrogenase (GAPDH).

### 2.5 Western blotting

For signalling targets, muscles were homogenised in ice-cold homogenisation buffer using clean sharp scissors. Samples were centrifuged at 11,000 g for 10 min at 4^°^C, and the supernatant was removed and quantified by Nanodrop. Extraction of VDR proteins required homogenisation and preparation in a hyperosmolar lysis buffer (HLB) (urea 6.7 M, glycerol 10%, Tris–HCl 10 mM, SDS 1%, DTT 1 mM, PMSF 1 mM and protease inhibitor cocktail tablet (Roche, West Sussex, UK)) as previously described [28]. All samples were diluted in homogenisation buffer and 1× Laemmli loading buffer to the same concentration.

Samples were loaded onto a Criterion XT Bis-Tris 12% sodium dodecyl sulphate polyacrylamide gel electrophoresis (SDS-PAGE) gel (Bio-Rad, Hemel Hempstead, UK) for electrophoresis for 1 h at 200 V. Separated proteins were transferred onto a polyvinylidene difluoride (PVDF) membrane for 45 min at 100 V, then blocked in 5% low-fat milk in Tris-buffered saline and 0.1% Tween-20 (TBST) for 1 h at room temperature. Membranes were then incubated at 4^°^C overnight in 5% milk diluted in TBST primary antibody solutions. Afterward, membranes were washed 3 × 5 min with TBST and incubated for 1 h at room temperature in their respective horseradish peroxidase (HRP) conjugated secondary antibody, anti-rabbit (Cell Signalling Technologies) 1:2,000 5% low-fat milk in TBST or anti-mouse (Cell Signalling Technologies) 1:2,000 5% low-fat milk in TBST. Membranes were washed 3 × 5 min in TBST and incubated for 5 min in enhanced chemiluminescence reagent (Millipore, Watford, UK) and visualised using a Chemidoc XRS. Bands were quantified using ImageLab software and normalised to total loaded protein visualised by Coomassie brilliant blue staining [29]. Primary antibodies against p-AKT Ser473 (1:2,000, #4060), Pan-AKT (1:2,000, #4685), p-TSC2 Thr1462 (1:2,000, #3611), TSC2 (1:2,000, #4308), p-mTOR Ser2448 (1:2,000, #2976), mTOR (1:2,000, #2972), p-p70S6K1 Thr389 (1:2,000, #9234), p70S6K1 (1:2,000, #2708), p-S6RP Ser235/236 (1:2,000, #2211), S6RP (1:2,000, #2217), p-4e-BP1 Thr37/46 (1:2,000, #2855), 4e-BP1 (1:2,000, #9644), p-eIF4E Ser209 (1:2,000, #9741), eIF4E (1:2,000, #9742) were from Cell Signalling Technologies. Primary antibodies against VDR (D-6) (1:2,000, SC-13133) were from Santa Cruz.

### 2.6 Immunofluorescence and co-localisation

Five-micrometre-thick muscle cross-sections were cut at −22^°^C using a Cryostat, mounted on glass slides before air-drying at room temperature. Sections were fixed in acetone/ethanol (3:1) for 5 min, before washing three times in phosphate-buffered saline (PBS).

Fibre CSA, VDR expression and co-localisation analysis was undertaken at the University of Birmingham. For CSA analysis, primary antibodies toward VDR (Rabbit, Ab109234, Abcam) and dystrophin (Mouse, MANDYS1(3B7), Developmental Studies Hybridoma Bank, Iowa City) were diluted in 5% goat serum in PBS 1:50 and 1:200, respectively. Antibodies were applied to each section and incubated for 2 h in a humidity chamber at room temperature before being washed in PBS three times. Secondary fluorescent anti-rabbit (Alexa Fluor® 594, A11012, Invitrogen) and anti-mouse (Alexa Fluor® 488, A21121, Invitrogen) antibodies were diluted in PBS 1:200 and sections incubated for 30 min in a humidity chamber at room temperature. Slides were subsequently washed three times in PBS, and a 1:1,000 4′,6-diamidino-2-phenylindole (DAPI) stain (Invitrogen) in PBS applied for 5 min before three further PBS washes. Mounting media (Invitrogen) was applied to each section and dried in darkness overnight. Additional sections were probed using anti-MHC IIa (SC-71) or anti-MHC IIb (BF–F3) diluted 1:50 in PBS. Sections were subsequently imaged in a blinded fashion using a Nikon Eclipse E600 and analysed using ImagePro 3D capture software. Three random fields of view were measured per section. For mTOR and LAMP2 co-localisation, primary antibodies toward mTOR (#05–1592, Millipore) and LAMP2 (AP1824d, Abgent) in 5% goat serum in PBS at 1:200 and 1:100, respectively. Secondary fluorescent antibodies anti-mouse IgGγ1 (Alexa Fluor® 594, A11005) and anti-rabbit (Alexa Fluor® 488, A11034) were additionally used as stated before.

Subsequent analysis for of PAX7+ satellite cells (SCs) was undertaken at Örebro University. Briefly, muscle cross-sections were mounted and air-dried as previously described. Sections were incubated with primary antibody towards PAX7 (Developmental Studies Hybridoma Bank) before incubation with a biotinylated anti-mouse secondary antibody (BA-9200, Vector Laboratories) as described in (Bankolé et al., 2013).

### 2.7 RNA-seq analysis

RNA was extracted using an RNeasy mini kit (Qiagen), following the manufacturer's recommendations. All RNA samples had RIN [30] scores of greater than 8. RNA was prepared using the Tru-Seq RNA library preparation kit (Illumina). RNA-sequencing was carried out by Edinburgh Genomics using the Illumina HiSeq 4000 platform generating 75 bp paired end reads. After initial quality control and base calling, tag data were examined with Fast QC [31] and adaptor sequences trimmed where necessary using trimmomatic [32]. Unpaired reads were found to be of low quality and were dropped from the analysis. No set of paired reads failed quality control. Alignment and feature counts were generated using the Rsubread package in R [33]. Differential expression was examined using the edgeR package [34]. The count data was filtered as recommended by the authors of edgeR by identifying the count-per-million (CPM) at a count of 10 [35] and subsequent normalisation was obtained with the trimmed mean of M-values method [36]. Differential expression was analysed using the glmFit function of edgeR with design matrices to account for biological pairing between treated and control limbs. Subsequent gene-set testing was carried out using the GSEABase library [37] in R using gene-sets from the Molecular Signatures Database maintained by the Broad Institute [38]. PathVisio v 3.3.0 [39,40] was used to construct all pathway analysis, utilising pathways from the WikiPathways [41] repository. The *Rattus norvegicus* Derby Ensembl 91 database was used for identity mapping of genes, with log-fold changes (</> 0.26) of each gene mapped to pathway nodes and significantly altered genes (*p* < 0.05) visualised.

### 2.8 Human participants

Muscle samples from the Derby resistance exercise training (RET) study [42] underwent additional analyses. Ethical approval for the original study and subsequent analysis was granted by The University of Nottingham Medical School Ethical Advisory Committee. Male and female healthy participants aged 18–75 were recruited (n = 37, 23 male, 14 female 48.4y±2.6 y), ensuring confounding age-associated decreases in VDR expression and response to RET were reduced. All subjects trained three times a week for 20 weeks, with sessions lasting approximately 60 min. Training intensity was 70% 1-repetition max (RM), based on single sets of 12 repetitions with 2-min rests between sets of seated chest press, lat pull down, seated lever row, leg extension, seated leg curl, seated leg press, back extension and abdominal curls. Biopsies of *vastus lateralis* muscle were taken pre-RET regime, and post-RET biopsies were taken 3–7 days after the final training session before microarray analysis (Geo accession GSE47881, by Affymetrix Human Genome U133 Plus 2.0 Array, VDR NM_000376). DXA measurements were taken pre-/post-RET. Thirty-seven participants from whom both pre- and post-training plasma samples were available were included from the original study, with samples taken 3–7 days before and after the RET period. Participants trained throughout the year and received no Vitamin D supplementation.

### 2.9 Plasma 25[OH]D measures

Vitamin D plasma levels were assessed by liquid chromatography tandem mass spectrometry (LC-MS/MS), measuring both 25 [OH]D_2_ and 25 [OH]D_3_ before combining the values for a total 25 [OH]D value. Deuterated ^2^H_3_-25 [OH]D_2_ and ^2^H_3_-25 [OH]D_3_ internal standards in ethanol were added to each plasma sample before addition of ice-cold methanol and incubation at 4^°^C for 10 min. Heptane was subsequently added, and vortex mixed for 30 s. The heptane layer containing 25 [OH]D_2_, 25 [OH]D_3_ and internal standards was evaporated to dryness, resuspended in acetonitrile: ddH2O (65:35). Samples were run against a standard curve of known concentrations.

### 2.10 Statistical analysis

The results are shown as mean ± SEM. All analysis was performed by an unpaired (or paired where appropriate) t-test for two group comparisons, a one-way analysis of variance (ANOVA) with Bonferroni post-hoc analysis between multiple groups and linear regression for correlations on GraphPad Prism7. A *P* value less than 0.05 was considered to represent statistical significance.

## 3 Results

### 3.1 *In vivo* VDR-OE stimulates muscle fibre hypertrophy via enhanced translational efficiency and capacity

**Figure 1:**
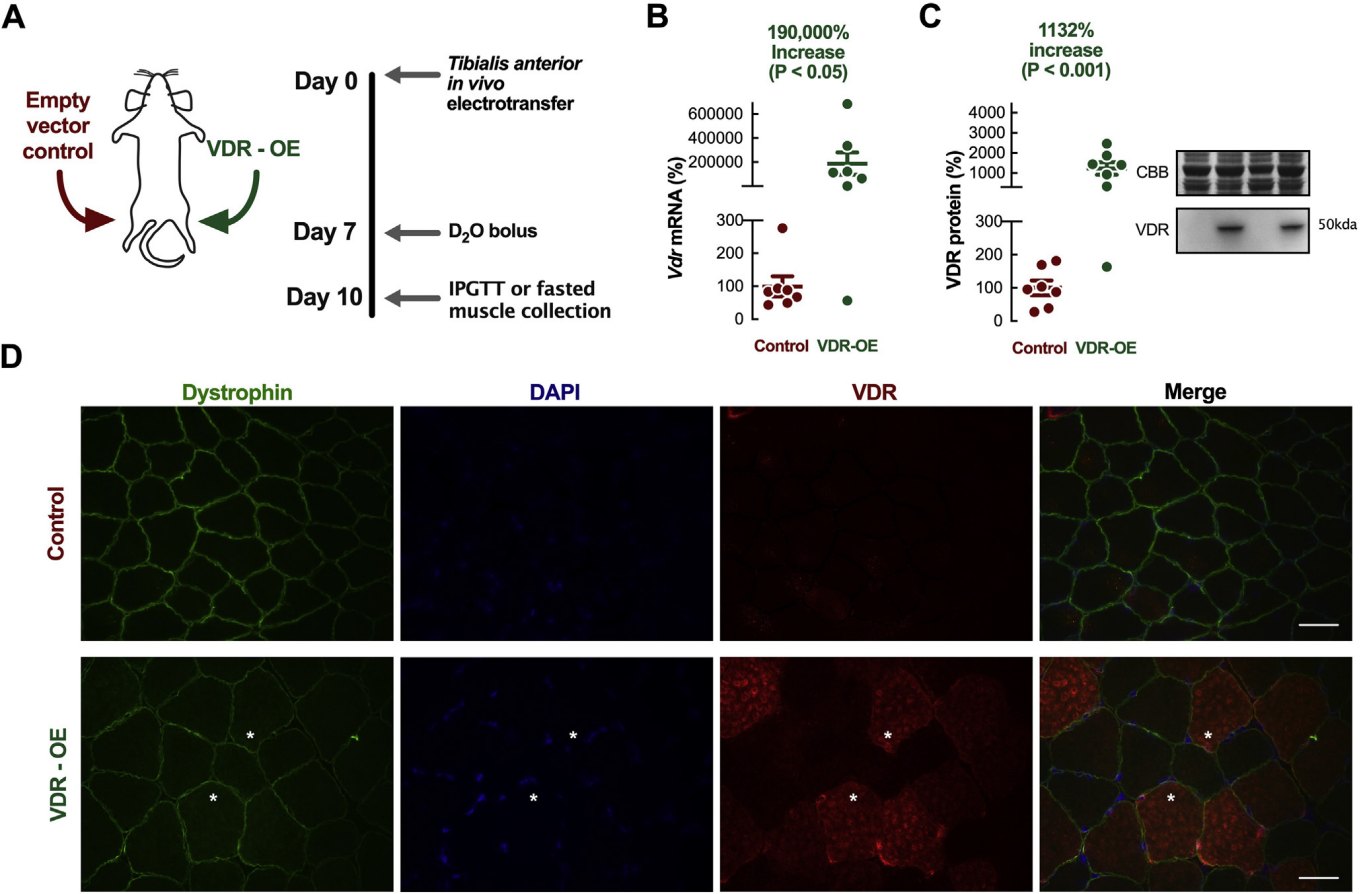
***In vivo* experimental design and grouping.** (A) Schematic design of *in vivo* paired contralateral experiments. (B) Confirmation of contralateral VDR-OE by qRT-PCR (n = 7). (C) Representative western blot and quantification of VDR-OE (=7n). (D) Representative images of muscle fibres stained for dystrophin (green), DAPI (blue) and VDR (red). Scale bars represent 200 μm. Data are mean ± SEM. Significance indicated measured by paired t-test. (For interpretation of the references to color in this figure legend, the reader is referred to the Web version of this article.)

**Figure 2:**
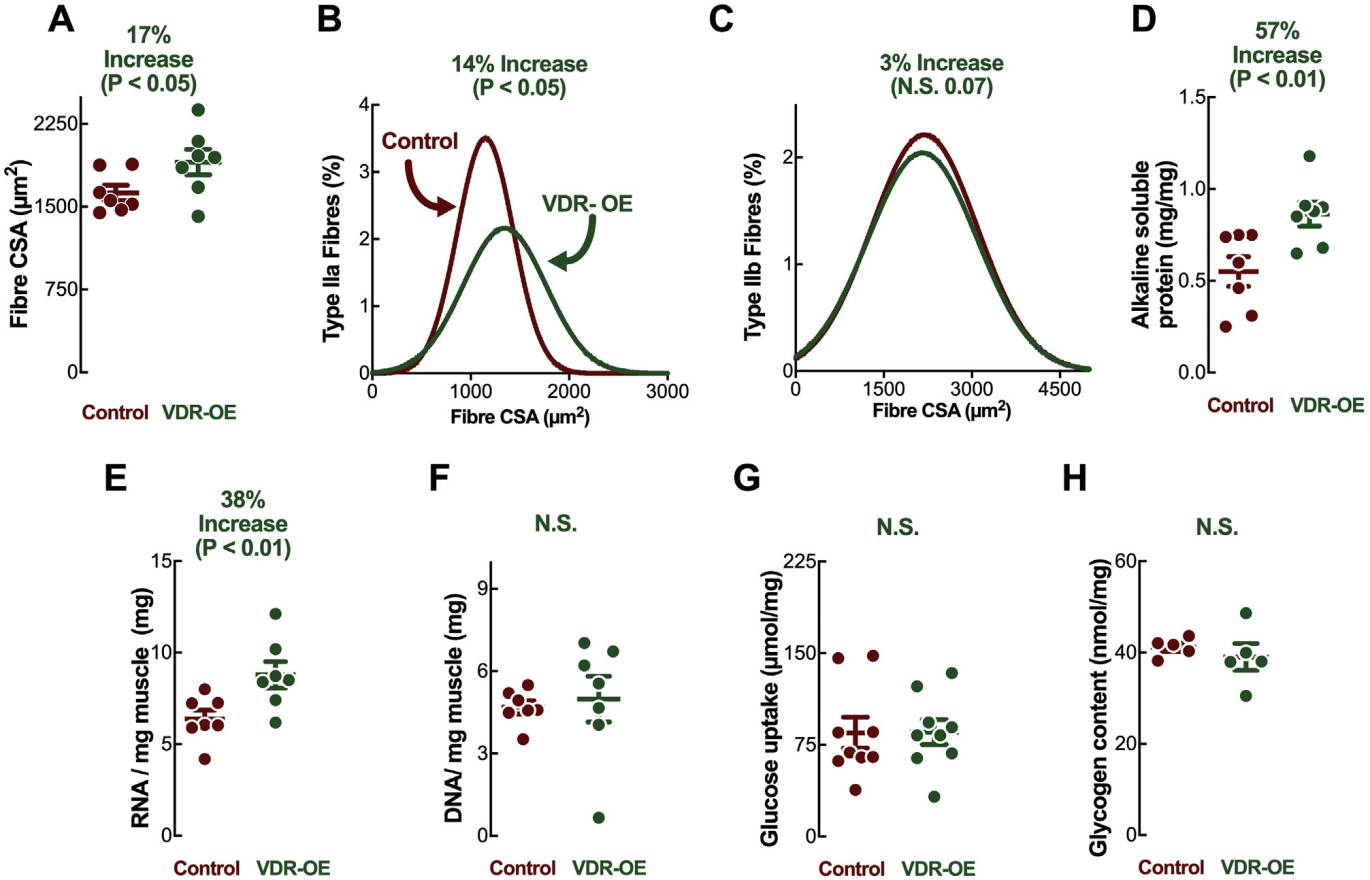
***In vivo* VDR-OE results in muscle fibre hypertrophy.** (A) All fibre CSA analysis, (B) Type IIa and (C) IIb fibre CSA distribution. Three random fields of view were measured per section in both L and R TA muscles in each animal (n = 7), with CSA measured for all intact fibres. (D) Alkaline soluble protein measures, (E) RNA and (F) DNA quantification per mg of dried muscle (n = 7). (G) Glucose uptake measured by ^3^H—2-deoxyglucose tracer uptake (n = 9). (H) Muscle glycogen content (n = 5). Data are mean ± SEM. Significance by paired t-test.

To examine the muscle-specific role of the VDR, we employed *in vivo* electrotransfer (IVE) to induce intracellular uptake of DNA constructs injected into rat *tibialis anterior* muscles [43], causing VDR gain of function (overexpression) (VDR-OE) (Figure 1A). Basal VDR expression was confirmed at both the mRNA and protein level, albeit at low levels similar to previous investigations [12]. VDR-OE was observed at the mRNA level (VDR-OE: +190,000 ± 9,000%) and the protein level (VDR-OE: +1,232 ± 311%) after 10 days (Figure 1B,C) at supraphysiological levels, similar to previous instances of IVE [23]. Additionally, sarcoplasmic VDR-OE was clearly visible in myofibres when immuno-stained using the anti-VDR antibody (Figure 1D). Moreover, VDR-OE evoked marked increases in myofibre CSA (Figure 2A) (VDR-OE 1,904 ± 116 μm^2^ vs. Control 1,627 ± 69 μm^2^, *P* < 0.05*)*, predominantly in fast type IIa fibres (VDR-OE 1,393 ± 17 μm^2^ vs. Control 1,218 ± 18 μm^2^, *P* < 0.05*)* (Figure 2B,C) in comparison to contralateral control limbs. This was coupled to a greater protein content in VDR-OE (VDR-OE 0.86 ± 0.07 mg vs. Control 0.55 ± 0.08 mg, *P* < 0.01, Figure 2D), which also coincided with a greater total RNA content per unit muscle (VDR-OE 8.79 ± 0.72 mg vs. Control 6.39 ± 0.47 mg, *P* < 0.05, Figure 2E), suggestive of enhanced ribosomal biogenesis. Despite these increases, total DNA content (Figure 2F) and glucose uptake/glycogen content (Figure 2G,H) per unit of muscle were unaffected after 10 days of VDR-OE.

**Figure 3:**
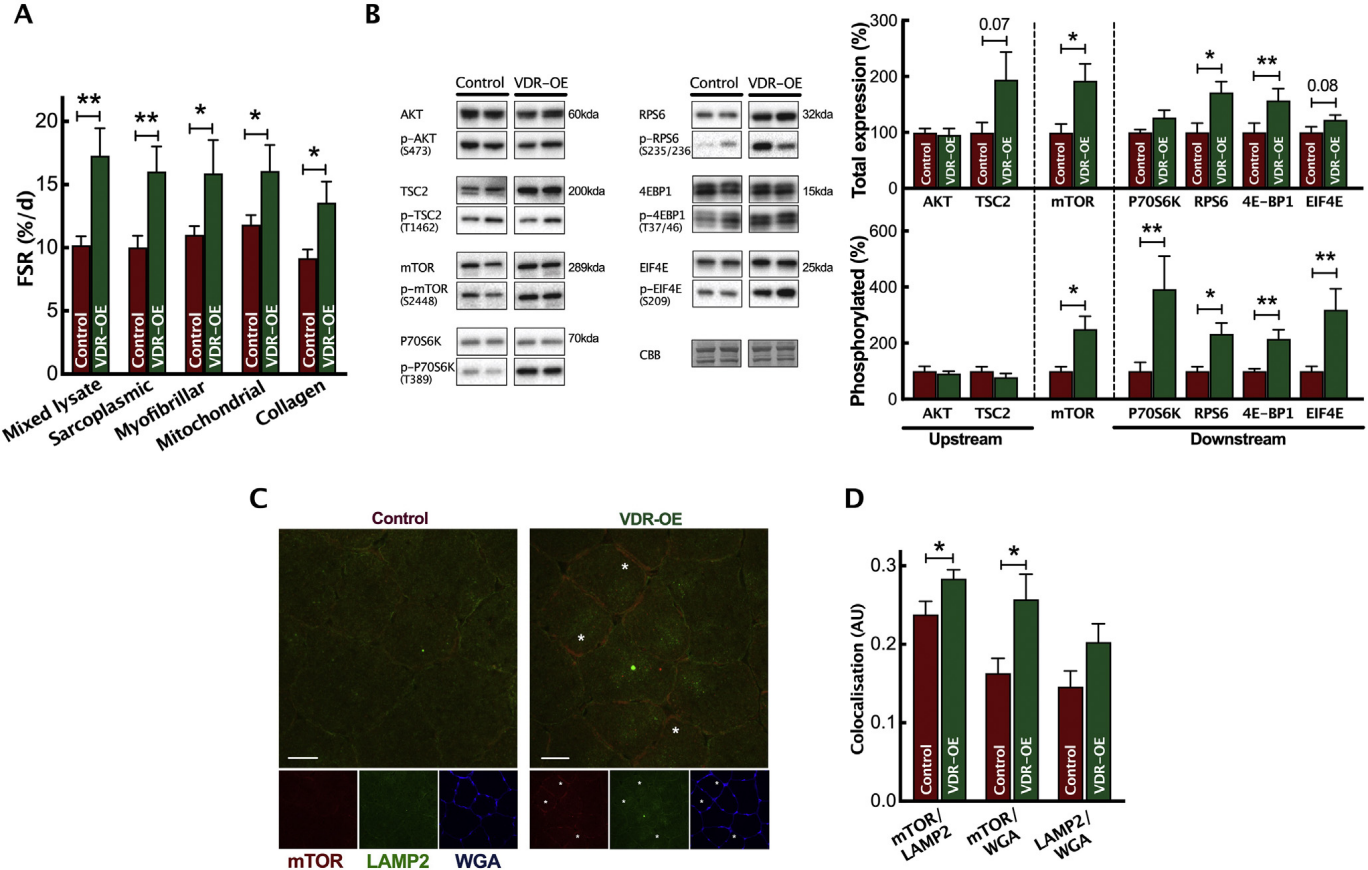
***In vivo* VDR-OE increases anabolic signalling and translational efficiency.** (A) Measurement of MPS rates of mixed lysate, sarcoplasmic, myofibrillar, mitochondrial and collagen protein subfractions by D_2_O incorporation (n = 7). (B) Representative western blots and quantification of phosphorylated and total protein anabolic signalling intermediates (n = 7). CBB, Coomassie Brillian Blue. (C) Representative images of mTOR and LAMP2 co-localisation. (D) Quantification of mTOR and LAMP2 co-localisation (n = 7). Data are mean ± SEM. ∗*p* < 0.05, ∗∗*p* < 0.01 between indicated groups. (For interpretation of the references to color in this figure legend, the reader is referred to the Web version of this article.)

Given the marked protein accretion with VDR-OE, we next determined the extent to which changes in post-transcriptional protein catabolism or anabolism were responsible for myofibre hypertrophy. In doing so, we observed a significantly higher global (i.e., total mixed muscle lysate) MPS rate in VDR-OE muscles than in control muscles (VDR-OE 17.3 ± 2.2 %d vs. Control 10.2 ± 0.7 %d, *P* < *0.01)* (Figure 3A). This was reflected in the systematic upregulation of individual sarcoplasmic, myofibrillar, mitochondrial and structural collagen fractions. To investigate the molecular underpinnings of VDR-OE-mediated increases in MPS, we undertook analysis of AKT-mTORc1 signalling pathways (essential for the upregulation of MPS [44,45]) and demonstrated increased phosphorylation and expression of multiple AKT/mTORc1 signalling intermediates (Figure 3B). VDR-OE enhanced protein expression of mTOR, along with that of multiple downstream (i.e., RPS6, 4E-BP1), but not upstream (i.e., AKT/TSC2), signalling intermediates. To confirm the enhanced activation of mTOR, immunohistochemical staining was undertaken to study the co-localisation of mTOR and lysosome-associated membrane protein 2 (LAMP2) (VDR-OE 0.26 ± 0.03 AU vs. Control 0.16 ± 0.02 AU, *P* < 0.05, Figure 3C,D). Following VDR-OE, there was significantly greater mTOR localisation with LAMP2, consistent with mTOR localisation at the lysosome, which is a pre-requisite for its activation [46].

**Figure 4:**
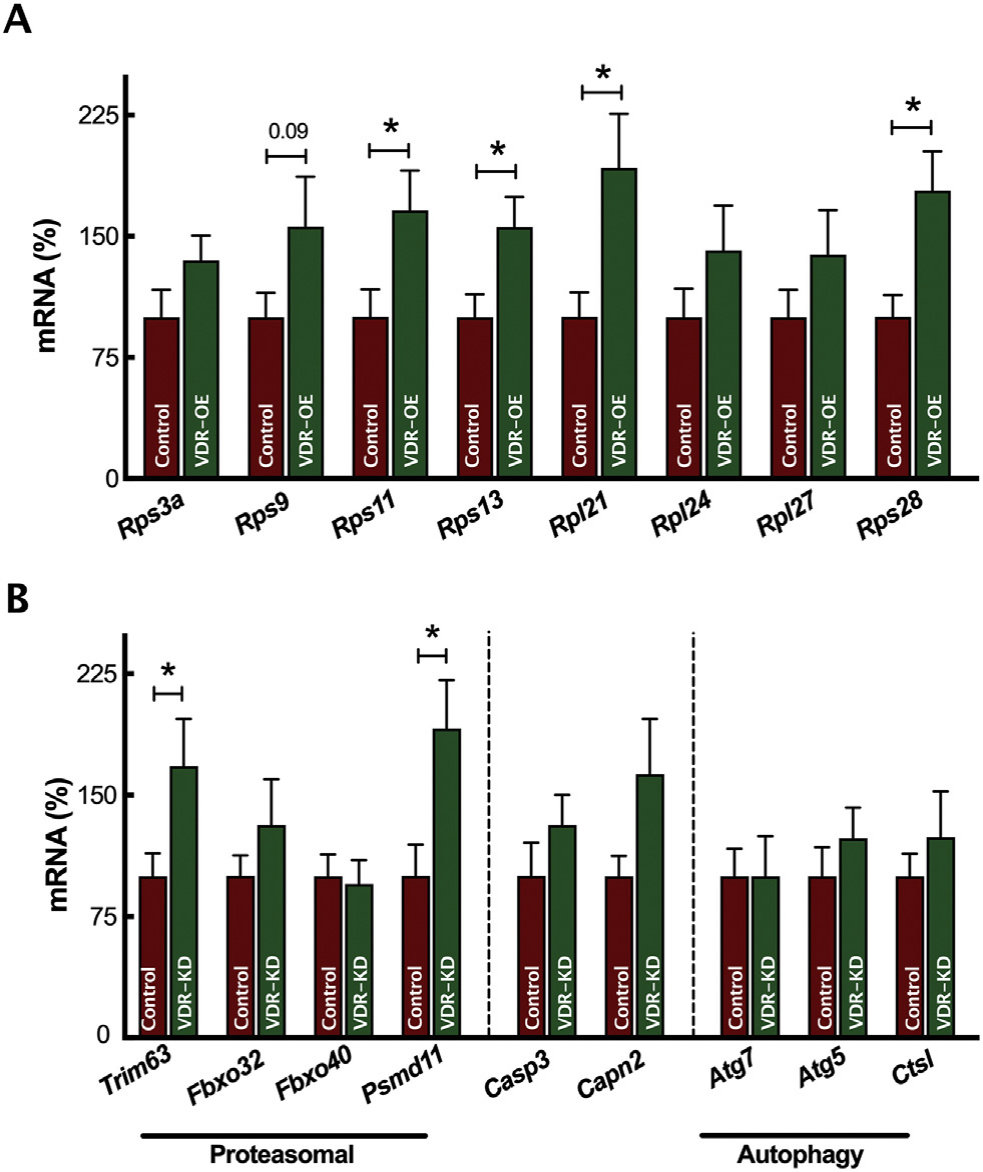
***In vivo* VDR-OE enhances ribosomal biogenesis.** qRT-PCR measurement of (A) ribosomal and (B) proteolytic related gene expression (n = 7). Data are mean ± SEM. ∗*p* < 0.05 between indicated groups.

Cellular growth and mTOR activation also requires heightened ribosomal biogenesis [47]. In support of this, total RNA content (per mg muscle) was higher in VDR-OE muscles, reflecting a greater synthetic capacity [48]. Furthermore, qRT-PCR screening of multiple ribosomal genes, demonstrated significant increases in the expression of numerous ribosomal short (e.g., *Rps11* VDR-OE 177 ± 26% vs. Control 100 ± 17%, *P* < 0.05, Figure 4A) and long genes (e.g., *Rps21* VDR-OE 192 ± 34% vs. Control 100 ± 18%, *P* < 0.05) following VDR-OE. Importantly, screening of multiple proteolytic markers indicated no downregulation within autophagic- or caspase-mediated degradation but demonstrated increased expression of proteasomal-mediated degradation markers (e.g., *Trim63* VDR-OE 168 ± 29% vs. Control 100 ± 9%, *P* < 0.05, Figure 4B).

### 3.2 RNA-Seq analyses demonstrate VDR-OE stimulates remodelling processes

**Figure 5:**
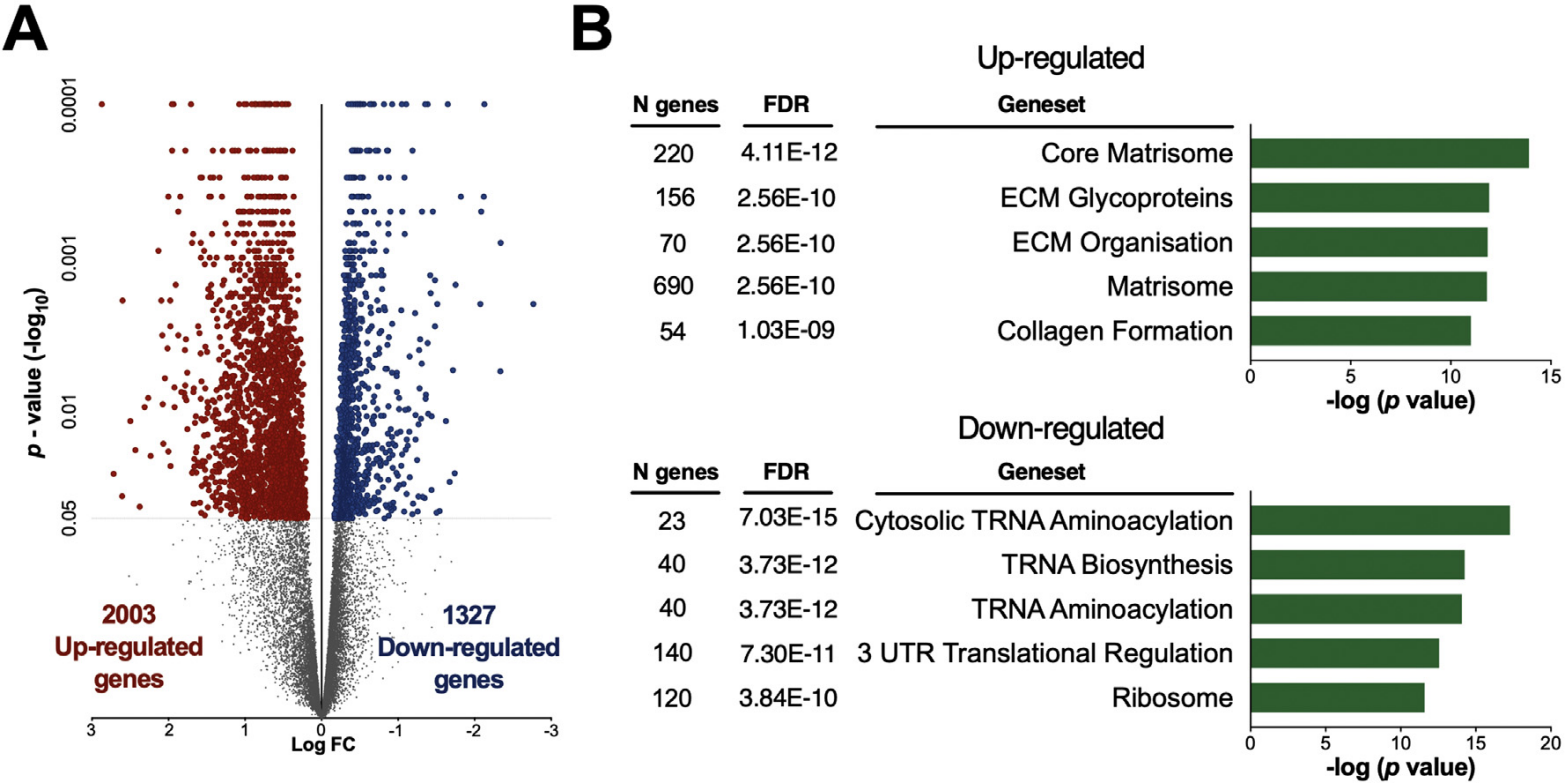
**VDR-OE upregulates hypertrophy and extracellular remodelling related gene-sets.** (A) Volcano plot of *p* < 0.05 statistically significant up-/downregulated genes. (B) Top five upregulated and downregulated gene-sets from the molecular signatures database in VDR-OE muscles (n = 7). See also Supplemental File 1.

Initial RNA-Seq analysis demonstrated that VDR-OE was associated with altered expression of 3,330 genes (*P* < 0.05) (Figure 5A) and numerous gene-sets (n = 310, FDR <5%) (Supplemental file 2), particularly relating to the extracellular matrix (ECM) (Figure 5B), including multiple gene-sets directly tied to ECM remodelling (i.e., NABA_CORE_Matrisome (*P* = 1.24E-14), NABA_ECM_Regulators, *P* = 2.56E-09), collagen formation (i.e., Reactome_Collagen formation, *p* = 1.01E-11), integrin-mediated signalling (i.e., PID_Integrin1 pathway, *P* = 2.82E-11) and KEGG_ECM receptor interaction, *P* = 1.80E-09) consistent with hypertrophic remodelling and growth. In contrast, VDR-OE also induced notable downregulation of gene-sets related to tRNA aminoacetylation (i.e., Reactome_Cytosolic tRNA aminoacetylation, *P* = 5.29E-18) and anabolic signalling gene-sets (i.e., BIOCARTA_mTOR pathway, *p* = 1.71E-05). Additional transcription factor (TF) analysis demonstrated upregulation of Pax- (i.e., Pax08_01 *p* = 0.0015, Pax04_01 *p* = 0.017) and serum response factor (SRF)- (i.e., SRF_01 *p* = 0.0027, SRF_Q4 *p* = 0.021) related gene-sets (Supplemental File 2).

**Figure 6:**
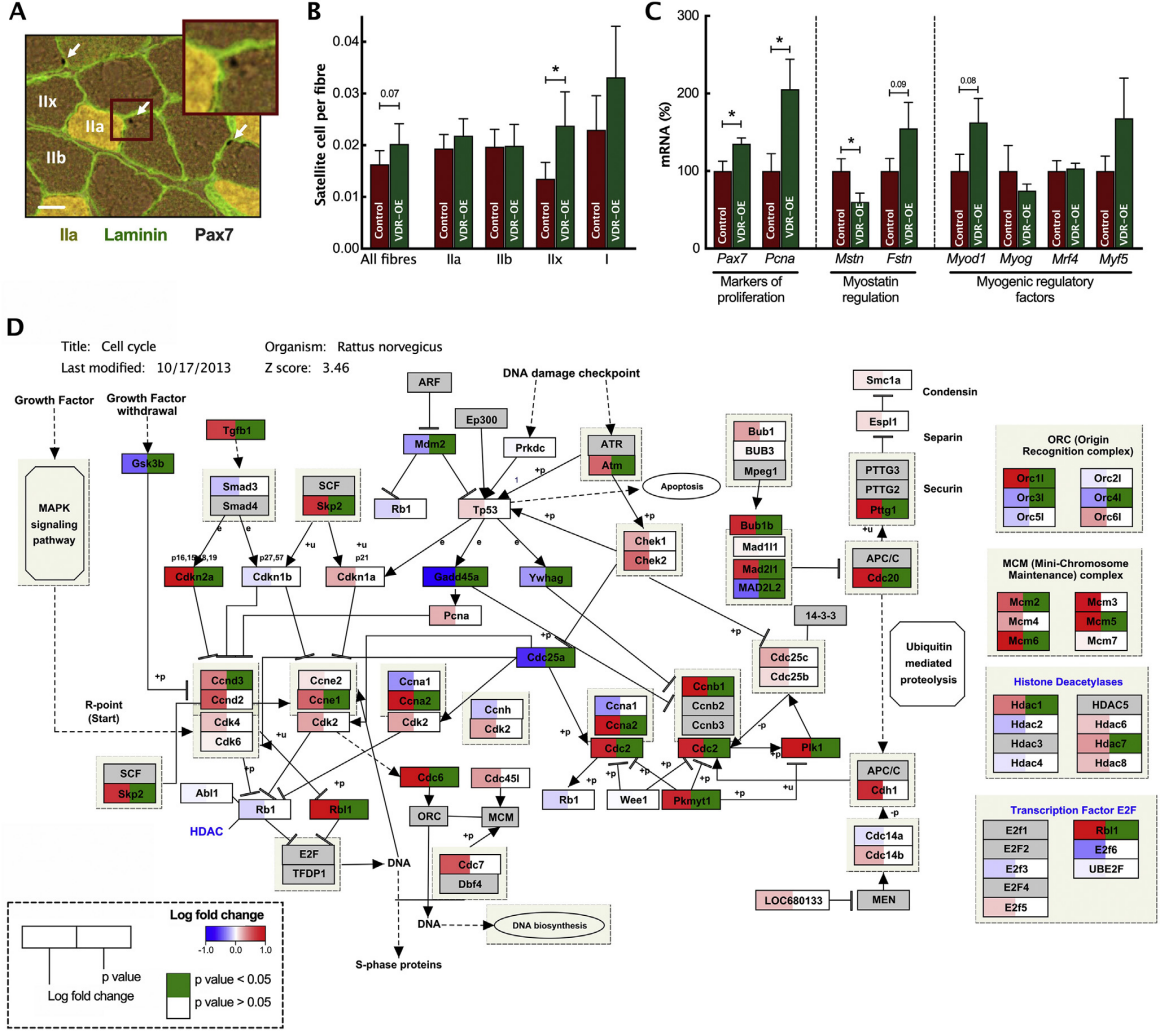
***In vivo* VDR-OE results in increased satellite cell content.** (A) Representative image of muscle fibres stained for Pax7 (Black), Laminin (Green) and IIa fibres (Yellow). Arrows signify Pax7+ nuclei of satellite cells and subsequent quantification (B). (C) qRT-PCR measurement for markers of proliferation and MRFs (n = 7). (D) RNA-Seq pathway analysis of *Rattus norvegicus* cell cycle gene expression. Log fold changes are shown as a gradient from red (upregulated) to blue (downregulated). *P*-values <0.05 are displayed as green. Scale bars represent 200 μm. Data are mean ± SEM. ∗*p* < 0.05, ∗∗*p* < 0.01 between indicated groups. (For interpretation of the references to color in this figure legend, the reader is referred to the Web version of this article.)

### 3.3 VDR-OE increases satellite cell recruitment

Given the crucial role of satellite cells (SC) in skeletal muscle growth, we next assessed paired box protein 7 (PAX7, expression required in proliferating satellite cells) immunochemical staining of VDR-OE muscles (Figure 6A), which showed a tendency for higher satellite cell content in all fibre types (VDR-OE 0.020 ± 0.004 per fibre vs. Control 0.016 ± 0.003 per fibre, *P* = 0.07, Figure 6B). While no alteration in fibre type distribution was observed, VDR-OE IIx fibres displayed a greater SC content than contralateral controls (VDR-OE 0.024 ± 0.007 per fibre vs. Control 0.014 ± 0.006 per fibre, *P* < 0.05). To assess the influence of VDR-OE upon satellite cell proliferation and content, we screened the expression of multiple myogenic genes (Figure 6C). (mRNA) *Pax7* expression was increased (VDR-OE 1.35 ± 0.07 FC vs. Control 1.00 ± 0.13 FC, *P* < 0.05), reflecting the increase in PAX7 protein. In addition to this, *Pcna expression*, a marker of satellite cell entry into the cell cycle [49], was higher in VDR-OE muscles (VDR-OE 2.1 ± 0.4 FC vs. Control 1.00 ± 0.2 FC, *P* < 0.05). Furthermore, *Mstn expression,* a known inhibitor of PAX7 and skeletal muscle hypertrophy [50], was reduced markedly (VDR-OE 0.6 ± 0.1 FC vs. Control 1.00 ± 0.2 FC, *P* < 0.05), which was accompanied by a moderate increase in *Fstn* (a myostatin inhibitor) expression (VDR-OE 1.6 ± 0.3 FC vs. Control 1.00 ± 0.2 FC, *P* = 0.09). We further measured the expression of multiple myogenic regulatory factors (MRFs) (Figure 6C). Interestingly, proliferative MRF genes (i.e., *Myod1* and *Myf5*) tended to be more highly expressed, whereas MRFs key to differentiation (i.e., *Myog* and *Mrf4*) [51] were unchanged. Additional RNA-Seq pathway analysis demonstrated an extensive upregulation in multiple cell cycle-related genes (Figure 6D), highlighting the widespread positive upregulation of cell proliferation. Taken together, these results indicate greater SC activity following VDR-OE, which is in agreement with the upregulation of *Pax* TF observed with RNA-Seq analysis (Supplemental File 2).

### 3.4 *VDR* expression, but not Vitamin D status or HOMA-IR, correlates with muscle hypertrophy in humans

**Figure 7:**
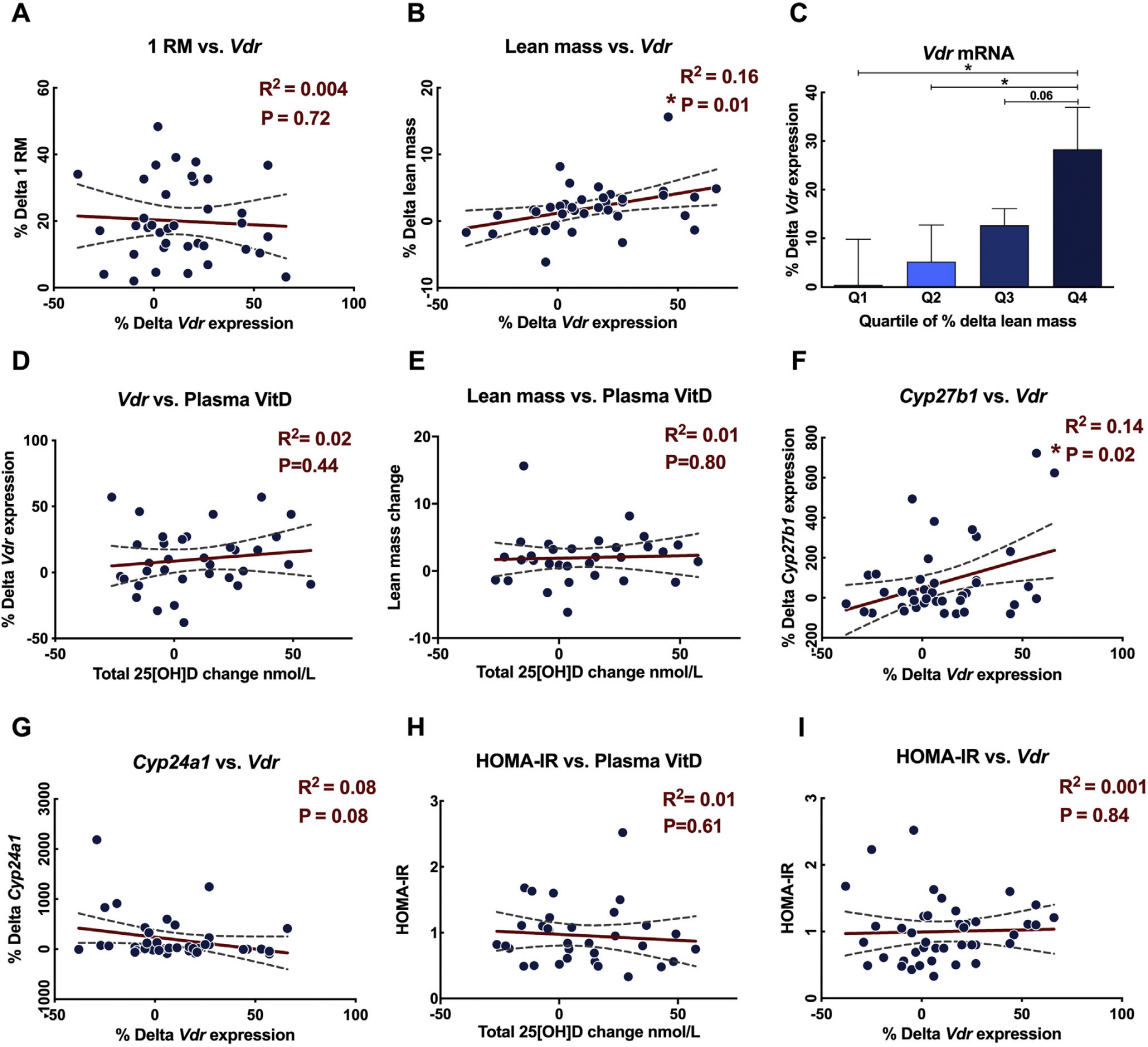
**VDR expression in humans correlated with increases in lean mass from RET.** (A) % Δ 1-RM vs. % Δ *Vdr* (n = 37). (B) % Δ Lean mass vs. % Δ *Vdr* (n = 37). (C) VDR expression in quartile groups of changes in lean mass. (D) % Δ 1-RM vs. Δ Plasma Vitamin D (n = 37). (E) % Δ Lean mass vs. Δ Plasma Vitamin D (VitD) (n = 37). (F) % Δ *Cyp27b1* vs. % Δ *Vdr* (n = 37). (G) % Δ *Cyp24a1* vs. % Δ *Vdr* (n = 37). (H) Post training HOMA-IR vs. Δ Plasma VitD (n = 37). (H) Post training HOMA-IR vs. % Δ *Vdr* (n = 37). (J) Representation of VitD metabolism. All % Δ changes are between pre- and post-training. Column data are mean ± SEM. ∗*p* < 0.05 between indicated groups.

To determine the relevance of increased muscle VDR expression in relation to human muscle mass, we quantified *VDR* expression in humans (18-75y, n = 37) who had undertaken 20-weeks of whole-body resistance exercise training (RET) but did not receive Vitamin D supplementation. Average Pre and post RET Vitamin D (25 [OH]D) plasma was 42.4 ± 3.1 and 51.2 ± 3.5 nmol/l, respectively (Supplemental data). Fasted-resting *Vastus lateralis* muscle biopsies and blood samples were taken before and after the training program; gene expression was measured by microarray analysis [42]. While *VDR* expression did not correlate with changes in strength (Figure 7A), lean mass gains positively correlated with changes in *VDR* gene expression (Figure 7B). Furthermore, when lean mass changes were plotted in quartiles, “responder status” for muscle hypertrophy tracked with *VDR* expression (Q1 0.4 ± 9.3% vs. Q4 28.3 ± 8.7%, *P* < 0.05, Figure 7C) (with no differences in VDR expression observed between quartiles pre-RET (data not shown)).

As *VDR* expression is correlated with Vitamin D exposure, we investigated changes in fasted circulating Vitamin D status (total 25-hydroxyvitamin D) and muscle *VDR* expression; however, no correlation was observed (Figure 7D). Additionally, no links were observed between plasma Vitamin D and lean mass gains (Figure 7E). However, changes in the gene expression of the Vitamin D handling enzyme *CYP27B1* displayed a positive correlation with VDR (Figure 7F), as did the expression of the Vitamin D-inactivating enzyme *CYP24A1,* which displayed a moderate negative correlation with *VDR* expression (Figure 7G). Finally, insulin sensitivity (HOMA-IR index) responses to RET was unrelated to Vitamin D status or muscle VDR expression (Figure 7H,I), consistent with our pre-clinical findings (Figures 2G).

## 4 Discussion

Despite numerous relationships between Vitamin D deficiency and loss of-function of VDR and low muscle mass and loss of function [5], the mechanistic role of VDR overexpression was undefined. Here, we explored whether overexpression of the VDR, independent of manipulation of Vitamin D, would positively regulate muscle mass *in vivo*. Herein, we reveal that transient gain-of-function of the VDR in skeletal muscle, leads to muscle hypertrophy and extracellular remodelling ostensibly through the post-translational upregulation of mTORc1 signalling coupled with enhanced ribosomal biogenesis and satellite cell proliferation. Finally, clinical observations show translational relevance, with correlative analysis revealing VDR expression patterns in muscle, tracked with gains in lean mass in humans undertaking prolonged, supervised resistance exercise training over 20-weeks.

Our investigations demonstrate that overexpression of the VDR in skeletal muscle results in robust myofibre hypertrophy, alongside concurrent gains in protein content synonymous with muscle growth, with increased protein synthesis across muscle protein sub-fractions (i.e., myofibrillar, sarcoplasmic, mitochondrial and collagen). Fundamentally, this mirrors previous investigations whereby deletion of the VDR induced myofibre atrophy and loss of muscle strength [18,19] and where muscle-specific deletion of the VDR resulted in reduced whole-body lean mass and function [10]. Crucially, increases in MPS are indispensable for protein accretion, occurring mainly via AKT/mTORc1 signalling [44], with rapamycin-mediated inhibition of mTOR blocking increases in MPS, e.g., as seen in response to resistance exercise (RE) [45]. While no parallel studies exist to the present investigation in relation to VDR-OE, exogenous Vitamin D supplementation activates AKT/mTORc1 signalling, resulting in increases in MPS that are correspondingly inhibited by rapamycin [52]. Furthermore, Vitamin D enhances anabolic stimulation of insulin/leucine via AKT/mTORc1, increasing MPS in muscle cell culture and augmenting signalling capacity, increasing insulin receptor expression [53]. Recent investigations have also demonstrated that VitD deficient patients (<50 nmol/l) with lower back pain have reduced spinal muscle VDR expression, corrected in response to 5 weeks of VitD supplementation [54]. Moreover, the same investigators demonstrated heightened anabolic capacity through enhanced muscle p-AKT/AKT content in response to supplementation [55]. Notably, we demonstrated enhanced protein expression of mTOR, along with that of all downstream signalling intermediates in VDR-OE muscles. Furthermore, heightened expression of mTOR corresponded with greater lysosomal (i.e., LAMP2) and cell membrane colocalisation, akin to mTOR activation by amino acids [46] or RET [56]. Thus, given that Vitamin D exerts its effects through both transcriptional and post-translational actions [13,14], the mTOR signalling and subsequent enhancement of MPS may be autonomously VDR-mediated, enhancing anabolic signalling capacity. Importantly, protein accretion may also occur as a result of a reduction in muscle protein breakdown (MPB) alongside increased MPS [57]. Analysis of VDR-OE muscle indicated no decrease in proteolytic markers (i.e., autophagy and calpain), indicating that muscle hypertrophy ostensibly occurred through the enhancement of protein synthesis, rather than the suppression of protein breakdown [57]. Moreover, observed increases in proteasomal markers are consistent with hypertrophic growth models (e.g., functional overload), whereby both protein synthesis and proteasomal degradation increase, with MuRF1 expression required for proper growth and remodelling [58].

Efficient regulation of MPS requires coordination of anabolic signalling combined with translational capacity (i.e., ribosomal content) to facilitate hypertrophy [59]. It follows that VDR-OE increased AKT/mTORc1 signalling activity, in tandem with global rates of mRNA translation reflected in increased protein synthesis across muscle protein sub-fractions (i.e., myofibrillar, sarcoplasmic, mitochondrial and collagen). Response to hypertrophic RET corresponds to an induction of muscle RNA content and translational capacity [60]. As such, greater total RNA content in VDR-OE muscles in addition to an increase of numerous rRNA's (by targeted qRT-PCR) demonstrates systematic adaptations required for sustained and successful hypertrophic growth. This is further substantiated as phosphorylation of ribosomal S6K1 via mTORc1-signalling, controls ribosomal biogenesis through induction of ribosomal biogenesis genes [61]. Moreover, recent investigations have determined that the VDR directly binds to multiple ribosomal 40s and 60s proteins, regulating transcriptional activity [62]. Finally, the downregulation of ribosomal gene-sets (by RNA-Seq analysis), in the presence of markedly upregulated total RNA pools, confirm a previously reported paradox that successful muscle growth is associated with down-regulation of *“growth”* gene-sets [42] when growth has been successful in high-responders to RE training. While initially appearing contradictory, RNA-Seq analysis requires specific library preperation using poly(A) capture, whereby highly abundant ribosomal RNAs and smaller species (<200 nucleotides) are selectively removed (accounting for >80% of total RNA) [63]. Importantly, both increased synthetic capacity (ribosomal content) and activity (signalling) demonstrate systematic increases, promoting a positive environment for sustained trophic growth in response to VDR-OE.

To facilitate hypertrophy, a robust upregulation of the muscle ECM is a characteristic transcriptional response, which in this study was echoed by our observation increased collagen protein synthesis. For instance, during synergist ablation-mediated hypertrophy, global changes in the muscle transcriptome paired with broad increases in anabolic signalling proteins evident across the time-course of muscle growth [64]. Moreover, ECM-related gene expression is heightened, with notable enhancement of basement membrane collagens assisting in the transmission of force in muscle [64]. Here, we used RNA-Seq analysis to demonstrate that ECM, integrin and structural gene-sets to be among the most upregulated, notably including enrichment of the core matrisome; a collection of genes encoding fundamental ECM proteins, including ECM glycoproteins, collagens and proteoglycans [65]. In skeletal muscle, the ECM provide structural support to growing fibres and enables mechanical transmission [66] via integrin-mediated signalling and focal adhesion complexes, vital to downstream AKT/mTORc1 anabolic signalling in response to muscle contraction [67,68]. Previous investigations have identified 3,000 VDR-target genes, with 50% being transcriptionally regulated by Vitamin D including matrisome and ECM-associated genes [69]. This regulation is recapitulated in VDR knockdown primary human skeletal muscle cells, as exogenous VitD induces VDR dependant systemic upregulation of focal adhesion, integrin signalling and ECM-related gene-sets [70]. Together, our findings indicate the VDR is at the nexus of ECM gene regulation, which may permit more effective contractile stimulated anabolic signalling and is likely a robust biomarker of protein accretion and hypertrophy.

Satellite cells (SCs) are indispensable for the regeneration of skeletal muscle [50,71] and likely have a mechanistic role in muscle hypertrophy through provision of myonuclei for fusion into pre-existing myofibres [72]. Here, we showed that VDR-OE was sufficient to enhance SC proliferation *in vivo,* as indicated by an increase in the recruitment of PAX7-positive cells [73], particularly in fast type IIX fibres. In support of this, further probing of RNA-Seq via TF (supplementary file 2) and pathway analysis demonstrated that VDR mediated upregulation of key SC regulatory genes, e.g., SRF (a regulator of SC proliferation and recruitment through paracrine signalling [74]) and multiple *Pax* genes. Recent investigations have demonstrated antipodal influences, whereby myocyte deletion of the VDR in mice results in systemic downregulation of cell cycle-related genes [10]. This is further substantiated by a reciprocal comprehensive up-regulation in cell cycle pathway genes. Potential regulatory functions of VitD upon cell cycle pathways have previously been observed in THP-1 cells. Thus, while stimulation of the VDR by VitD provides a positive modulatory role upon proliferation, it is likely to occur due to the positive role of increased VDR expression [75]. Similarly, past work has shown that VDR expression increases in skeletal muscle regenerating from injury [12] and is co-localised to SCs (PAX7-positive nuclei) [76]. Further, muscle cell motility (essential for SC fibre infiltration) is improved by administration of low Vitamin D concentrations (10 nmol) in an *in vitro* wound model (i.e., myoblasts and fibroblasts) [77]. It is important to note that the enhanced satellite cell proliferation is unlikely to have participated in the demonstrated muscle hypertrophy within the relatively short duration of VDR-OE (10 days), as fusion to pre-existing fibres is not likely to have occurred because only proliferative MRFs were increased at this time. That said, these data support the notion that the VDR alone can regulate SC proliferation, an essential aspect of muscle regeneration, while our findings both corroborate and extend the mechanistic evidence that enhanced SC proliferation is a feature of VDR-OE-induced muscle hypertrophy.

Given that different VDR genotypes [78] and altered regulation of *VDR* expression in human muscle [6] have been associated with the control of muscle mass, we next investigated the relevance of our findings in relation to human muscle mass following 20 weeks of RET [42], specifically, correlative muscle VDR transcriptional responses. In a previous investigation, VDR protein expression was shown to increase in response to acute resistance exercise in rats [22]. Our RET study resulted in an upregulation of the *VDR*, and crucially, showed that the heterogeneity of the gains in lean mass to the hypertrophic stimulus were closely correlated with *VDR* upregulation. A relationship was also evident with muscle Vitamin D-handling enzymes; *CYP27B1* expression positively correlated with an upregulation of VDR, potentially regulating local control of Vitamin D metabolism [8,9]. Therefore, Vitamin D status alone may not regulate muscle mass/function, but rather, sufficient VDR expression may be required. This may explain the ambiguity of efficacy seen within some Vitamin D supplemental studies and muscle function in both elderly [79] and young healthy individuals, as often VDR expression is not measured [7,9]. Furthermore, disparities between VDR regulation and plasma VitD may be due to technical difficulties in the quantification of VitD metabolites (i.e., distinction between metabolites e.g., 25 [OH]D_3_ and 3-epi-25 [OH]D_3_ [80]), seasonal variance or there being no direct relationship in human skeletal muscle. Finally, previous investigations have proposed that VitD has a role in type 2 diabetes (T2D) and glucose metabolism, enhancing glucose transport in muscles [81], while other investigations have observed inverse correlations between plasma 25 [OH]D and Homeostatic Model Assessment of Insulin Resistance (HOMA-IR) in humans [82,83]. However, we saw no relationship between changes in VitD status and HOMA-IR. Likewise, glucose uptake and glycogen storage were unchanged in response to VDR-OE. This may be due to VitD-/VDR-mediated influences upon glucose stimulated insulin secretion, relative to glucose metabolism, as observed in Vitamin D deficient T2D rat models [84]. Moreover, continual reduction in serum insulin concentrations following an OGTT has been described in VDR mutant mice [85]. This indicates that if VitD/VDR does have a role in glucose homeostasis, our models may be insufficient to detect this, or it could be mediated through effects on insulin secretion (not measured in our model), rather than glucose uptake in skeletal muscle.

There are also limitations within this study. These *in vivo* data are limited to the TA muscle, a predominantly type II fibre muscle used during the *in vivo* measures. While previous investigations have demonstrated faster type II-prominent muscle hypertrophy to a greater extent than slower type-I muscles (i.e., soleus), heightened MPS and fibre CSA increases consistent responses between muscle types [86]. Moreover, differences between baseline characteristics of differing muscle types is an important consideration, with ribosomal content in soleus muscles exceeding that of faster muscles. Thus, VDR regulated mechanisms of hypertrophy may differ between metabolically distinct muscles. While robust VDR upregulation is observed in response to RET, correlative analysis is unable to determine causative mechanisms; thus, further investigation is required to elucidate these. Furthermore, while direct comparisons between *in vivo* and correlative human data should be cautiously undertaken, we demonstrate coordination of overarching principles of muscle hypertrophy through regulation of MPS and ribosomal expansion. Finally, we demonstrate *in vivo* increases in anabolic signalling capacity and activation (total and phosphorylated respectively). VDR manipulation may influence how muscle responds to the stimulation of these pathways by diet (i.e., amino acids) or exercise. These data provide intriguing insight into the autonomous role of the VDR in skeletal muscle health and maintenance. Moreover, future studies may be able to elucidate the role of VDR within established anabolic stimuli, such as acute feeding responses or mechanical loading. Thus, Vitamin D analogues or targeted therapies increasing VDR expression may be a potential manner in which to enhance anabolic responses to dietary intervention or exercise, which is of particular importance in age-related anabolic blunting and sarcopenic development.

In conclusion, we demonstrate a positive role of enhanced VDR expression on skeletal muscle, showing that gain of function of VDR autonomously stimulates hypertrophy associated with increased AKT/mTOR anabolic signalling, muscle protein synthesis, ribosomal biogenesis and satellite cell activation. VDR expression and Vitamin D processing enzymes are significantly correlated with RET-induced hypertrophy in humans. Based on these data, VDR expression may be a robust marker of the hypertrophic response to resistance exercise. Additionally, Vitamin D analogues that promote increased VDR expression and/or VDR targeting therapies could be further developed to enhance and maintain skeletal muscle mass and responsiveness to exercise.

## Author contributions

JJB, KS, NJS, MEC and PJA designed the experiments. JJB, DA and MEC carried out *in vivo* sample collection. JJB, AN, CSD DJW, JT and FK performed data collection JJB, MSB, DJW, BEP, AP, JT, FK, KS, IJG, NJS, MEC and PJA analysed the data. JJB undertook RNA extraction for RNA-SEQ, with IJG performing bioinformatic analysis. JJB and AMG constructed the pathway analysis. JJB, MSB, DJW and KS performed mass spectrometry analysis. All authors contributed to the preparation and drafting of the manuscript.
