# Supplementary material for: Overexpression of the vitamin D receptor (VDR) induces skeletal muscle hypertrophy
Source: Mol Metab. 2020 Aug 7;42:101059. doi: 10.1016/j.molmet.2020.101059 (PMC7475200; doi:10.1016/j.molmet.2020.101059)
Supplement: Multimedia component 3 [file mmc3.docx]

**Supplementary Files**

**Supplemental File 1** - RNA-Seq analysis of gene expression in VDR-OE muscle compared to contralateral controls.

**Supplemental File 2** – Transcription factor gene set enrichment analysis in VDR-OE muscle compared to contralateral controls

**Supplemental Figure 1**


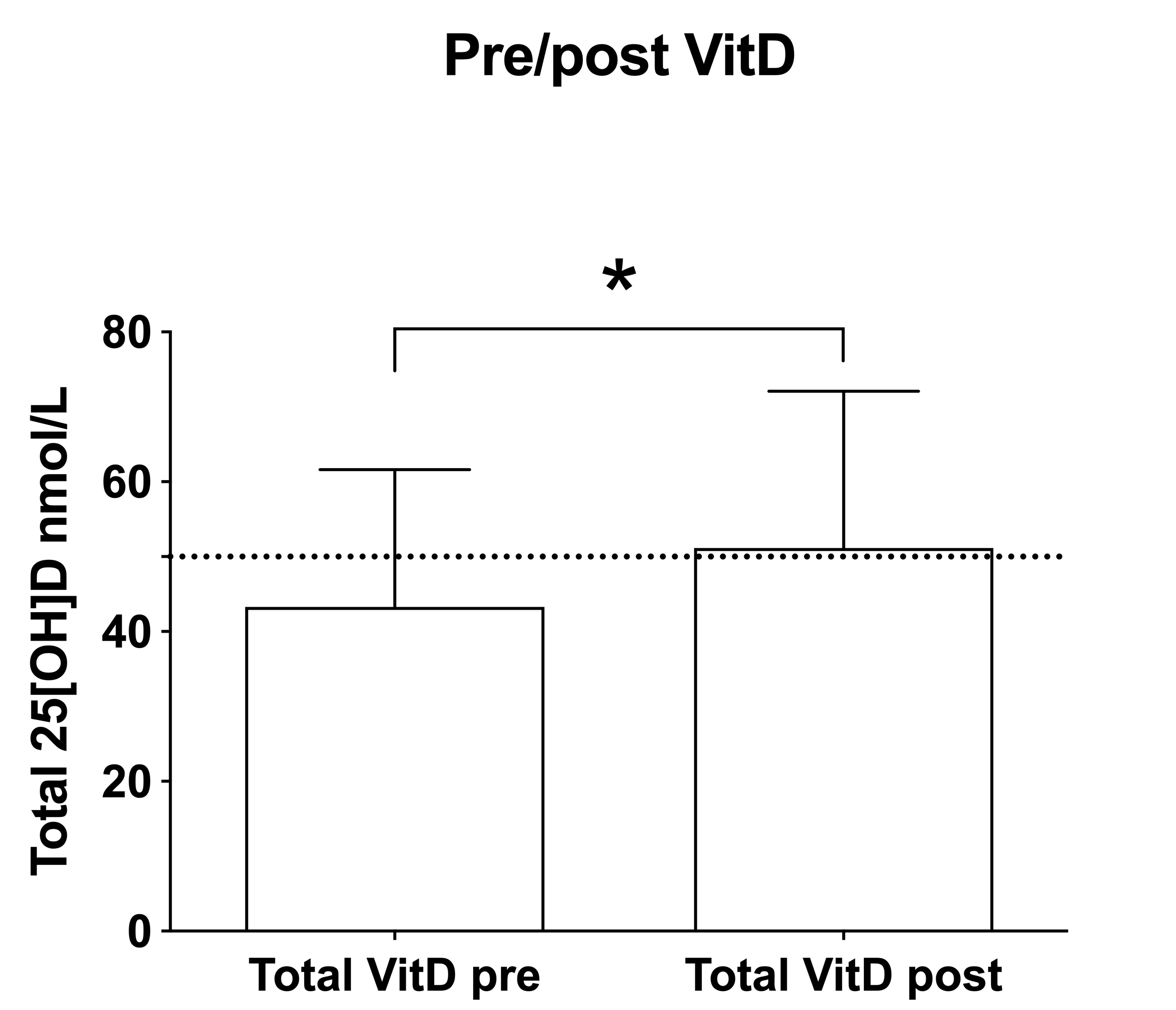


**Supplemental Figure 1**

Mean total plasma 25[OH]D (D2/D3 combined) pre and post 20-week resistance exercise training regime. Dashed line denotes Vitamin D deficiency of 50 nmol/l. Data are mean ±SD. **p*<0.05 between indicated groups.

**Table S1. qRT-PCR primers**

| **Gene** | **Accession number** |  | **Primer sequence** |
| --- | --- | --- | --- |
| **Rat** |  |  |  |
| GAPDH (Housekeeper) | NM_017008.4 | F | ATCCCGCTAACATCAAATGG |
|  |  | R | GTGGTTCACACCCATCACAA |
| VDR | NM_017058.1 | F | GGTTTCTTCAGGCGGAGCAT |
|  |  | R | GGTGATGCGGCAATCTCCAT |
| 4E-BP1 | NM_053857.2 | F | CCCGGGAGGAACCAGAATC |
|  |  | R | TCATCGCTGGTAGGGCTAGT |
| EIF4E | NM_053974.2 | F | AAACAAACGGGGAGGACGAT |
|  |  | R | CACAGCAGTGTCTCTAGCCAA |
| RPS6KB1 | NM_031985.1 | F | GCCTGTCAGCCCAGTCAAAT |
|  |  | R | AGAGTTGGGCTGTCGGATTG |
| RPS6 | NM_017160.1 | F | GCGCCTGCTTTTGAGTAAGG |
|  |  | R | TCCACAATGCATCCTCGGAC |
| RPL21 | NM_053330.1 | F | TAGACATCAAGGGAATGGGCAC |
|  |  | R | TTGTTCACAATGATGCCCACG |
| RPL24 | NM_022515.1 | F | CGATGGGAAGGTTTTCCAGT |
|  |  | R | ATATCAGCCAGAGAAGCGCC |
| RPL27 | NM_022514.1 | F | GCTGTCGAAATGGGCAAGTT |
|  |  | R | GTCGGAGGTGCCATCATCAA |
| RPS3a | NM_017153.1 | F | GTGGCAGACCATGATTGAAGC |
|  |  | R | TTGTCTGCACTTCTCGGGTC |
| RPS9 | NM_031108.4 | F | GTCACGGGCCTGAGTTTGTA |
|  |  | R | CCTCCACACCTCACGTTTGT |
| RPS11 | NM_031110.1 | F | TGTTTCAGGGACGTCCAGATT |
|  |  | R | GAGCATTGGCTACAGTCCCC |
| RPS13 | NM_130432.2 | F | TCTCTCTTTGCTTCCAGACCT |
|  |  | R | ATCACACCTATCTGGGAAGGAG |
| RPS28 | NM_001105730.1 | F | CCATCAAGCTGGCTAGGGTAA |
|  |  | R | TCTCGAACAGGGCCTTTGAC |
| Fstn | NM_012561.1 | F | GTGTGCCATGAAGGAAGCTG |
|  |  | R | TCCGAGATGGAGTTGCAAGA |
| Mrf4 | NM_013172.2 | F | TAAGGAAGGAGGAGCAAGCG |
|  |  | R | GGGAGTTTGCGTTCCTCTGA |
| Myf5 | NM_001106783.1 | F | TCAAACGCATGTGCTTCAGAT |
|  |  | R | GGTTGCAGGCTGTGAATTGG |
| Myod1 | NM_176079.1 | F | CTGCTCTGATGGCATGATGG |
|  |  | R | CTCCACTATGCTGGACAGGC |
| Myog | NM_017115.2 | F | GTGAATGCAACTCCCACAGC |
|  |  | R | CGAGCAAATGATCTCCTGGGT |
| Mstn | NM_019151.1 | F | ACCATGCCTACCGAGTCTGA |
|  |  | R | ATCCACAGCTGGGCCTTTAC |
| Pax7 | NM_001191984.1 | F | AGCCGAGTGCTCAGAATCAA |
|  |  | R | TCCTCTCGAAAGCCTTCTCC |
| Pcna | NM_022381.3 | F | GCAACTTGGAATCCCAGAACA |
|  |  | R | AAGGTCCCGGCATATACGTG |
| Trim63 (Murf1) | NM_080903.1 | F | CACCTTCCTCTTGAGTGCCA |
|  |  | R | CTCAAGGCCTCTGCTATGTGT |
| Fbxo32 (Atrogin-1) | NM_133521.1 | F | AGCTTGTGCGATGTTACCCA |
|  |  | R | GGTGAAAGTGAGACGGAGCA |
| Fbxo40 | XM_006248404.3 | F | CGGGGTTGGCATAAGTGCTA |
|  |  | R | CAGAGGACCCGAGTTGACTTC |
| PSMD11 | NM_001107027.1 | F | CGACCCAATCATCAGCACAC |
|  |  | R | GGCCTCTTACCAGACAGACAG |
| ATG5 | NM_001014250.1 | F | CAGAAGCTGTTCCGTCCTGT |
|  |  | R | CCGTGAATCATCACCTGGCT |
| ATG7 | NM_001012097.1 | F | CAGCCTGTTCATCCAAAGTTCTTG |
|  |  | R | CTGTGGTTGCTCAGACGGT |
| Ctsl | NM_013156.2 | F | CTATCGCCACCAGAAGCACA |
|  |  | R | ACCACACTGGCCCTGATTCT |
| Casp3 | NM_012922.2 | F | CGGACCTGTGGACCTGAAAA |
|  |  | R | CGGCCTCCACTGGTATCTTC |
| Capn2 | NM_017116.2 | F | TCGGCATCTATGAGGTCCCA |
|  |  | R | ATTCTTGTGGGGCTCGAAGG |
